# Allogeneic hematopoietic cell transplantation for T-cell/histiocyte-rich large B-cell lymphoma: An EBMT lymphoma working party study

**DOI:** 10.1038/s41375-026-02993-4

**Published:** 2026-06-15

**Authors:** Simon Renders, Maud Ngoya, Laurence de Leval, Herve Finel, Irma Khvedelidze, Rachael Pocock, Ron Ram, Tobias Gedde-Dahl, Yasmina Serroukh, Gesine Bug, Nicolaus Kröger, Arnon Nagler, Thomas Pabst, Werner Rabitsch, Ibrahim Yakoub-Agha, Matthias Eder, Siret Ratip, Christoph Scheid, Jose Antonio Pérez-Simón, Eva Maria Wagner-Drouet, Anna Sureda, Norbert Schmitz, Bertram Glass, Ali Bazarbachi, Peter Dreger

**Affiliations:** 1 Heidelberg University Hospital, Department of Medicine V, Hematology, Oncology and Rheumatology, Heidelberg, Germany; 2 German Cancer Research Center (DKFZ) and German Cancer Consortium (DKTK), Heidelberg, Germany; 3 Heidelberg Institute for Stem Cell Technology and Experimental Medicine (HI-STEM gGmbH), Heidelberg, Germany; 4 Lymphoma Working Party, European Society for Blood and Marrow Transplantation Central Registry Office, Paris, France; 5 Institute of Pathology, Department of Laboratory Medicine and Pathology, Lausanne University Hospital and Lausanne University, Lausanne, Switzerland; 6 University College London Hospitals NHS Foundation Trust, London, UK; 7 BMT Unit, Tel Aviv Sourasky Medical Centre, and Tel Aviv University, Tel Aviv, Israel; 8 Oslo University Hospital, Rikshospitalet, Oslo, Norway; 9 Erasmus UMC Cancer Institute, Department of Hematology, University Medical Centre, Rotterdam, The Netherlands; 10 Goethe University University Hospital Frankfurt, Dept. of Medicine 2, Frankfurt, Germany; 11 Universitätsklinikum Hamburg-Eppendorf, Hamburg, Germany; 12 Chaim Sheba Medical Center, Tel Hashomer, Israel; 13 Inselspital Bern, Bern, Switzerland; 14 Medical University of Vienna, Wien, Austria; 15 CHU de Lille, Univ Lille, INSERM U1286, Infinite, 59000 Lille, France; 16 Hannover Medical School, Hannover, Germany; 17 Acıbadem University, Istanbul, Turkey; 18 University Hospital of Cologne, Department of medicine I, Cologne, Germany; 19 Hospital Universitario Virgen Del Rocío, Instituto de Biomedicina de Sevilla (IBIS/ CSIC), Universidad de Sevilla, Sevilla, Spain; 20 University Hospital Mainz, Mainz, Germany; 21 Clinic Hematology Department, Institut Català d’Oncologia – L’Hospitalet, Institut d’Investigacions Biomèdiques de Bellvitge (IDIBELL), Universitat de Barcelona, Barcelona, Spain; 22 University Hospital Muenster, Münster, Germany; 23 HELIOS Klinikum Berlin-Buch, Berlin, Germany; 24 Bone Marrow Transplantation Program, Department of Internal Medicine, American University of Beirut, Beirut, Lebanon

**Keywords:** B-cell lymphoma, Stem-cell therapies

## Abstract

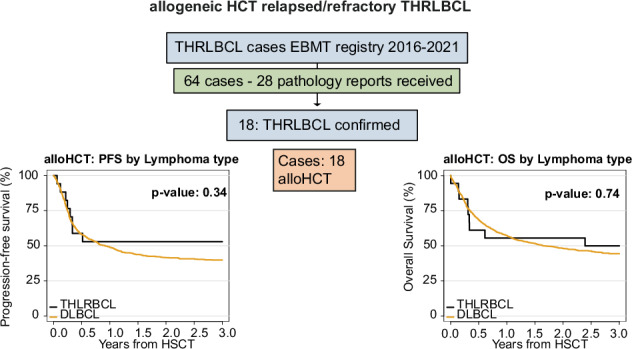

## To the Editor

T-cell/histiocyte-rich LBCL (THRLBCL) is an uncommon entity within the large B-cell lymphoma family, comprising less than 5% of all LBCL [1]. Compared to other LBCL, THRLBCL patients are younger, commonly male, present with higher International Prognostic Indexes (IPI), and advanced disease stages [2]. THRLBCL is named for its unique immunomodulating micro-milieu characterized by T-cell and histiocyte infiltration. Of note, THRLBCL overexpresses multiple immune checkpoint-molecules like PD-1, PD-L1 or LAG3. This mirrors Hodgkin lymphoma (HL) and a subfraction of patients with THRLBCL has a documented history of nodular lymphocyte-predominant Hodgkin lymphoma/ B-cell lymphoma (NLPHL/NLPBCL) [1, 3]. The histologic similarities with HL and peripheral T-cell lymphomas lead to common misdiagnosis and likely underreporting of the disease. Despite the diagnostic uncertainties and the clinical and biological peculiarities described, the prognosis of THRLBCL appears to be similar to that of the most common form of LBCL, diffuse LBCL not otherwise specified (DLBCL, NOS) if treated with DLBCL-typical first-line chemoimmunotherapy [2].

Nevertheless, a considerable proportion of patients with THRLBCL fails first-line therapy and needs salvage treatment. Apart from high-dose therapy with autologous hematopoietic cell transplantation (auto-HCT), cellular immunotherapies, specifically allogeneic HCT (allo-HCT) and CD19-targeting chimeric antigen receptor-engineered T-cell (CAR-T) therapies, have proven efficacy in the LBCL salvage setting [4–7]. While a few case series on CAR-T therapies in THRLBCL with mostly disappointing results have been published, information on allo-HCT in patients with THRLBCL is extremely limited.

Therefore, we studied real-world outcomes of allo-HCT in patients with r/r THRLBCL compared to DLBCL. Given the challenges associated with a correct histopathological diagnosis of THRLBCL, we primarily relied on cases with available pathology reports reviewed by an experienced hematopathologist and deemed consistent with THRLBCL.

Outcome data was extracted from the EBMT registry database. Informed consent for transplantation and data collection was obtained locally according to regulations applicable at the time of therapy. Eligibility criteria for this study were: Diagnosis of THRLBCL or DLBCL, first allo-HCT between 2016 and 2020, and age ≥18 years at cellular therapy. All patients identified in the EBMT database fulfilling these criteria and having a minimum baseline data set available (age, sex, diagnosis, date of diagnosis, date of cellular therapy, number of pretreatment lines) represented the study cohort. For all patients registered as THRLBCL, diagnostic reports were requested from the centers and reviewed by an experienced hematopathologist (LdL). Patients with reports not consistent with THRLBCL were excluded. Cases without available report were considered separately.

Primary endpoint was progression-free survival (PFS) 2 years after transplantation. Other endpoints analyzed were non-relapse mortality (NRM), relapse incidence (RI), and overall survival (OS). OS was defined as time from transplant to death from any cause, PFS as time from transplantation until disease relapse/progression or death from any cause. NRM included all causes of death without prior disease progression/relapse. Patient-, disease- and transplant-related variables were compared between cohorts using the Chi-square test or Fisher’s exact test for categorical variables and the Mann-Whitney test for continuous variables. The probabilities of OS and PFS were calculated using the Kaplan-Meier estimate. Cumulative incidences of RI and NRM were calculated accommodating for competing risks. Univariate analyses were performed on the matched dataset using log-rank comparisons for OS and PFS. The Gray test was used for cumulative incidence functions. Cox analysis was performed on the matched analysis to compare risk. All analyses were performed using R statistical software version 4.2.2 (available online at http://www.R-project.org) and IBM SPSS version 26.0 (SPSS Inc., Chicago, IL).

**Fig. 1:**
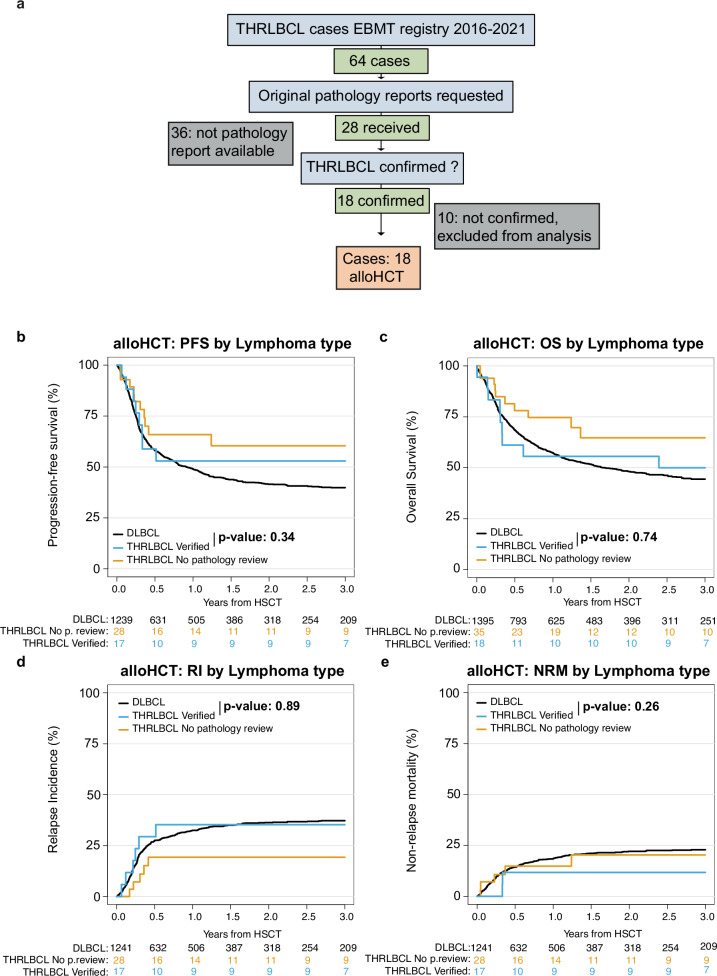
Outcome of pathology-reviewed and verified THRLBCL patients with allo-HCT. **a** Overview of cases selected for survival analysis after histological report review, Kaplan-Meier curves depicting **b** Kaplan-Meier curves depicting PFS, **c** OS, **d** RI and **e** NRM for verified THRLBCL and DLBCL patients after allo-HCT.

A total of 1476 patients with THRLBCL or DLBCL meeting the eligibility criteria were identified in the EBMT database. Of these, 64 had the diagnosis THRLBCL and 1,412 DLBCL. Diagnostic reports were received for 28 patients registered with THRLBCL from 19 centers. In 10 patients the reports were suggestive for lymphoma entities other than THRLBCL, or were not consistent with the diagnosis of THRLBCL for other reasons, leaving 18 patients with confirmed THRLBCL (Fig. 1a). Within the limits of heterogeneous available information contained in the reports, some of the cases appeared to correspond to THRLBCL-like evolution or transformation of NLPHL/BCL.

The baseline characteristics of the 18 allo-HCT recipients are summarized in Table 1. Of note, the vast majority proceeded to transplant with chemo-sensitive disease. With a median follow-up of 4.3 years, 6 relapse/progression and 2 NRM events were observed, all within the first the first 7 months after allo-HCT, resulting in 2-year PFS and OS rates of 53% (95%CI 28–73%) and 56% (95%CI 31–75%), respectively (Fig. 1b, c). Compared to 1412 DLBCL patients with similar baseline characteristics, except younger age and fewer previous lines of therapy in THRLBCL, no significant differences were detected in terms of PFS, OS, RI, and NRM (Table 1, Fig. 1b–e). Of note, in contrast to THRLBCL, relapse events continued to occur beyond the 6 months landmark in the DLBCL cohort (Fig. 1b,d).

**Table 1:** Baseline characteristics of pathology-reviewed and verified THRLBCL and DLBCL patients treated with allo-HCT.

		THRLBCL	DLBCL	*P-value*
		*N* = 18	*N* = 1412	
**Age patients (years)**	median (range)	41.3 (19.3-62.2)	53.4 (18-75.9)	***0.0003***
**Interval diagnosis to HCT**	>12 months	14 (77.8)	1016 (72)	0.5844
	0-12 months	4 (22.2)	396 (28)	
**Year of HCT**	median (range)	2018 (2016-2020)	2018 (2016-2020)	0.3115
**Sex patients**	Female	4 (22.2)	509 (36.1)	0.2235
	Male	14 (77.8)	902 (63.9)	
	missing	0	1	
**Sex donor**	Female	8 (44.4)	490 (35.2)	0.4163
	Male	10 (55.6)	901 (64.8)	
	missing	0	21	
**Donor type**	Matched relative	5 (27.8)	451 (32)	0.95
	Mismatched relative	3 (16.7)	260 (18.4)	
	Unrelated donor	10 (55.6)	700 (49.6)	
	missing	0	1	
**Karnofsky Index**	Good >=80	13 (86.7)	1205 (91.8)	0.3527
	Poor <80	2 (13.3)	107 (8.2)	
	missing	3	100	
**HCT Comorbidity Index**	0	12 (80)	668 (57.7)	0.2803
	1-2	1 (6.7)	231 (20)	
	>3	2 (13.3)	258 (22.3)	
	missing	3	255	
**International Prognostic Index (IPI-Score)**	0-2 points	1 (14.3)	352 (53.1)	0.2707
	3-5 points	6 (85.8)	311 (46.9)	
	missing	11	749	
**Disease status at HCT**	CR/PR	15 (83.3)	1078 (79)	1
	Progression/relapse	0 (0)	275 (20.1)	
	missing	3 (16.7)	59	
**Myeloablative regimen**	No	7 (38.9)	808 (59)	0.0847
	Yes	11 (61.1)	561 (41)	
	missing	0	43	
**Lines of treatment**	1	14 (77.8)	701 (49.6)	***0.0177***
	2 or more	4 (22.2)	711 (50.4)	
**HSC Source**	BM	0 (0)	117 (8.3)	0.3905
	PB	18 (100)	1295 (91.7)	
**Use of ATG/ALG**	ATG or ALG	7 (38.9)	591 (41.8)	0.8428
	No ATG or ALG	11 (61.1)	821 (58.1)	
**Use of PTCY**	Yes	4	347	Not done
	missing	14	1065	
**Conditioning Regimens**	Busulfan-based	7 (38.9)	535 (38.7)	Not done
	TBI-based	2 (11.1)	325 (23.5)	
	Other	9 (50)	525 (38.0)	
	missing	0	27	

Busulfan based (BuCy/BuFlu), TBI-based: TBI+Mel, TBI+CyFluThio+ other TBI based,

Other (BEAM, CyFlu, MelFlu, Treo-based, other), BEAM: Carmustin, Etoposide, Cytarabine, Melphalan;

*Cy* Cyclophosphamide, *Flu* Fludarabine, *Mel* Melphalan, *Threo* Threosulfan, *Bu* Busulfan, *Thio* Thiotepa,

*TBI* Total body irradiation ATG: Anti-thymocyte globulin, *ALG* Anti-lymphocyte globulin,

*PTCY* Post-transplant Cyclophosphamide, *CR* Complete remission, *PR* Partial remission, *PD* Progressive disease.

Characteristics and outcome of the patients with non-reviewed THRLBCL undergoing allo-HCT were not significantly different from those with verified pathology reports. However, a trend towards higher age was observed in the not-reviewed group. As young age is a feature of THRLBCL, this suggests a contamination of non THRLBCL cases also within the non-reviewed cases (Fig. 1b–e, Supplemental Table 1).

While there is abundant published experience with allo-HCT in DLBCL, even from the CAR-T era [5–8], specific information on the safety and efficacy of allo-HCT in patients with relapsed/refractory THRLBCL is virtually absent. This is particularly important as preliminary data including the evidence provided in this study suggest that cellular immunotherapy through CAR-T therapies is mostly associated with a remarkably poor efficacy [3, 9–12].

Here we show for the first time that allo-HCT is effective and feasible in patients with THRLBCL. With more than half of the patients living progression-free at the 2-year landmark, the efficacy of allo-HCT in THRLBCL appears to be at least as good as in DLBCL, where published outcomes are in line with those observed in this study, i.e. 2-year PFS rates in chemosensitive patients between 40% and 50% [5–8]. In contrast to our experience with auto-HCT [4], all THRLBCL relapse/progression events occurred early after allo-HCT, suggesting at least some contribution of graft-versus-lymphoma activity to long-term disease control.

The second major finding in this study is that high quality reporting on THRLBCL requires central expert review. In the absence of diagnostic tumor samples, review of pathology reports resulted in the exclusion of more than one third of cases registered. Similar drop-out rates have been reported previously for other rare lymphoma entities and to some extent reflect the difficulties encountered when interpreting results from registry studies on orphan or difficult-to-diagnose entities also beyond the lymphoma cosmos[13].

With the continuous advent of novel therapies for LBCL, the arsenal of options to induce responses before transplantation are increasing rapidly. Since the curative potential of antibody drug conjugates, e.g. Loncastuximab tesirine, or bispecific antibodies alone or in combinations remains uncertain [14, 15], the role of allo-HCT for consolidating targeted treatment responses of relapsed/refractory LBCL having failed or being ineligible for CAR T-cell therapy may undergo a renaissance.

In conclusion, allo-HCT appears to be a curative therapeutic option for a substantial proportion of patients with r/r THRLBCL responding to salvage therapy. In the absence of individual biopsies, central review of diagnostic reports appears to be strongly advisable in registry studies involving orphan diseases and those difficult to diagnose.

## Supplementary information

Supplemental Table 1 Legend

Supplemental Table 1

## References

[1] Campo E, Jaffe ES, Cook JR, Quintanilla-Martinez L, Swerdlow SH, Anderson KC, et al. The International Consensus Classification of Mature Lymphoid Neoplasms: a report from the Clinical Advisory Committee. Blood. 2022;140:1229–53. 35653592 10.1182/blood.2022015851 PMC9479027

[2] Ollila TA, Reagan JL, Olszewski AJ. Clinical features and survival of patients with T-cell/histiocyte-rich large B-cell lymphoma: analysis of the National Cancer Data Base. Leuk Lymphoma. 2019;60:3426–33. 31287335 10.1080/10428194.2019.1639166 PMC6928430

[3] Nair R, Ogundipe I, Gunther J, Medeiros LJ, Jain P, Nastoupil LJ, et al. Outcomes in Patients with Relapsed Refractory T-Cell/Histiocyte-Rich Large B-Cell Lymphoma Treated with CAR-T Cell Therapies or Salvage Chemotherapy - a Single-Institution Experience. Blood. 2023;142:6327–6327.

[4] Renders S, Ngoya M, Finel H, Rubio MT, Townsend W, Schroers R, et al. Autologous stem cell transplantation in T-cell/histiocyte-rich large B-cell lymphoma: EBMT Lymphoma Working Party study. Blood Adv. 2024;8:5571–8. 39213423 10.1182/bloodadvances.2024013152 PMC11541691

[5] Dreger P, Dietrich S, Schubert ML, Selberg L, Bondong A, Wegner M, et al. CAR T cells or allogeneic transplantation as standard of care for advanced large B-cell lymphoma: an intent-to-treat comparison. Blood Adv. 2020;4:6157–68. 33351108 10.1182/bloodadvances.2020003036 PMC7756983

[6] Dreger P, Sureda A, Ahn KW, Eapen M, Litovich C, Finel H, et al. PTCy-based haploidentical vs matched related or unrelated donor reduced-intensity conditioning transplant for DLBCL. Blood Adv. 2019;3:360–9. 30723110 10.1182/bloodadvances.2018027748 PMC6373757

[7] Fenske TS, Ahn KW, Graff TM, DiGilio A, Bashir Q, Kamble RT, et al. Allogeneic transplantation provides durable remission in a subset of DLBCL patients relapsing after autologous transplantation. Br J Haematol. 2016;174:235–48. 26989808 10.1111/bjh.14046 PMC4940282

[8] Derigs P, Bethge WA, Kramer I, Holtick U, von Tresckow B, Ayuk F, et al. Long-Term Survivors after Failure of Chimeric Antigen Receptor T Cell Therapy for Large B Cell Lymphoma: A Role for Allogeneic Hematopoietic Cell Transplantation? A German Lymphoma Alliance and German Registry for Stem Cell Transplantation Analysis. Transplant Cell Ther. 2023;29:750–6. 37709204 10.1016/j.jtct.2023.09.008

[9] Pophali PA, Fein JA, Ahn KW, Allbee-Johnson M, Ahmed N, Awan FT, et al. CD19-directed CART therapy for T-cell/histiocyte-rich large B-cell lymphoma. Blood Adv. 2024;8:5290–6. 38985302 10.1182/bloodadvances.2024013863 PMC11497379

[10] Trujillo JA, Godfrey J, Hu Y, Huang J, Smith SM, Frigault MJ, et al. Primary resistance to CD19-directed chimeric antigen receptor T-cell therapy in T-cell/histiocyte-rich large B-cell lymphoma. Blood. 2021;137:3454–9. 33881502 10.1182/blood.2020009148 PMC8212512

[11] Bastos-Oreiro M, Iacoboni G, Garces VN, Caballero AC, Martinez N, Delgado J, et al. Chimeric antigen receptor T-cell therapy outcomes in T cell/histiocyte-rich large B-cell lymphoma and subsequent treatment strategies after disease progression: A GELTAMO/GETH study. Hemasphere. 2025;9:e70077. 39906687 10.1002/hem3.70077 PMC11792173

[12] Jacobson CA, Hunter BD, Redd R, Rodig SJ, Chen PH, Wright K, et al. Axicabtagene Ciloleucel in the Non-Trial Setting: Outcomes and Correlates of Response, Resistance, and Toxicity. J Clin Oncol. 2020;38:3095–106. 32667831 10.1200/JCO.19.02103 PMC7499617

[13] Tanase A, Schmitz N, Stein H, Boumendil A, Finel H, Castagna L, et al. Allogeneic and autologous stem cell transplantation for hepatosplenic T-cell lymphoma: a retrospective study of the EBMT Lymphoma Working Party. Leukemia. 2015;29:686–8. 25234166 10.1038/leu.2014.280

[14] Caimi PF, Ai W, Alderuccio JP, Ardeshna KM, Hamadani M, Hess B, et al. Loncastuximab tesirine in relapsed or refractory diffuse large B-cell lymphoma (LOTIS-2): a multicentre, open-label, single-arm, phase 2 trial. Lancet Oncol. 2021;22:790–800. 33989558 10.1016/S1470-2045(21)00139-X

[15] Abramson JS, Ku M, Hertzberg M, Huang HQ, Fox CP, Zhang H, et al. Glofitamab plus gemcitabine and oxaliplatin (GemOx) versus rituximab-GemOx for relapsed or refractory diffuse large B-cell lymphoma (STARGLO): a global phase 3, randomised, open-label trial. Lancet. 2024;404:1940–54. 39550172 10.1016/S0140-6736(24)01774-4

