# Supplementary material for: Allogeneic hematopoietic cell transplantation for T-cell/histiocyte-rich large B-cell lymphoma: An EBMT lymphoma working party study
Source: Leukemia. 2026 Jun 15;40(8):1802–6. doi: 10.1038/s41375-026-02993-4 (PMC13421346; doi:10.1038/s41375-026-02993-4)
Supplement: Supplementary file 1 — Supplemental Table 1 Legend [file 41375_2026_2993_MOESM1_ESM.docx]

**Supplemental Tables**

***Supplemental Table 1: Baseline characteristics of THRLBCL patients treated with allo-HCT: Pathology-review verified vs. no pathology review***
