# Supplementary material for: Allogeneic hematopoietic cell transplantation for T-cell/histiocyte-rich large B-cell lymphoma: An EBMT lymphoma working party study
Source: Leukemia. 2026 Jun 15;40(8):1802–6. doi: 10.1038/s41375-026-02993-4 (PMC13421346; doi:10.1038/s41375-026-02993-4)
Supplement: Supplementary file 2 — Supplemental Table 1 [file 41375_2026_2993_MOESM2_ESM.pdf]

allo HCT: THRLBCL patients, pathology verified vs. no pathology review

		verified N = 18	no p. review N = 36	*P-value*
**Patients age (years)**	median (range)	41.3 (19.3-62.2)	48.7 (19.1-65)	0.1007
**Interval diagnosis to HCT**	>12 months	14 (77.8)	26 (74.3)	0.5206
	0-12 months	4 (22.2)	9 (25.7)	
	missing	0	1	
**Year of HCT**	median (range)	2018 (2016-2020)	2017 (2016-2020)	0.742
**Sex Patients**	Female	4 (22.2)	8 (22.2)	1
	Male	14 (77.8)	28 (77.8)	
**Sex donor**	Female	8 (44.4)	11 (31.4)	0.3494
	Male	10 (55.6)	24 (68.6)	
	missing	0	1	
**Karnofsky Index**	Good >=80	13 (86.7)	29 (87.9)	1
	Poor <80	2 (13.3)	4 (12.1)	
	missing	3	3	
**HCT Comorbidity Index**	0	12 (80)	25 (83.3)	0.4921
	1-2	1 (6.7)	4 (13.3)	
	>3	2 (13.3)	1 (3.3)	
	missing	3	6	
**International Prognostic Index (IPI-Score)**	0-2 points	1 (14.3)	11 (52.3)	0.1995
	3-5 points	6 (85.8)	10 (47.7	
	missing	11	15	
**Disease status at HCT**	CR/PR	15 (83.3)	31 (93.9)	0.3311
	Progression/relapse	3 (16.7)	2 (6.1)	
	missing	0	3	
**Myeloablative regimen**	No	25 (47.2)	18 (51.4)	0.3865
	Yes	28 (52.8)	17 (48.6)	
	missing	1	1	
**Lines of salvage treatment**	1	14 (77.8)	17 (47.2)	***0.0323***
	2 or more	4 (22.2)	19 (52.8)	
**HSC Source**	BM	0 (0)	7 (19.4)	0.0816
	PB	18 (100)	29 (80.6)	
**Use of ATG/ALG**	ATG or ALG	7 (38.9)	8 (22.2)	0.2944
	No ATG or ALG	11 (61.1)	28 (77.8)	
**Use of PTCY**	Yes	4	9	Not done
	missing	14	27	
**Conditioning Regimens**	Busulfan-based	7 (38.9)	17 (47.2)	Not done
	TBI-based	2 (11.1)	4 (11.1)	
	Other	9 (50)	15 (41.7)	

Busulfan based (BuCy/BuFlu), TBI-based: TBI+Mel, TBI+CyFluThio+ other TBI based,
Other (BEAM, CyFlu, MelFlu, Treo-based, other), BEAM: Carmustin, Etoposide, Cytarabine, Melphalan;
Cy: Cyclophosphamide; Flu: Fludarabine; Mel: Melphalan, Threo: Threosulfan; Bu:Busulfan; Thio: Thiotepa;
TBI: Total body irradiation ATG: Anti-thymocyte globulin; ALG: Anti-lymphocyte globulin;
PTCY:Post-transplant Cyclophosphamide, CR: Complete remission; PR: Partial remission, PD: Progressive disease
